# Cancer Niche as a Garbage Disposal Machine: Implications of TCM-Mediated Balance of Body-Disease for Treatment of Cancer

**DOI:** 10.33552/OJCAM.2019.01.000514

**Published:** 2019-06-11

**Authors:** Shengwen Calvin Li, Jane Luo, Katherine L Lee

**Affiliations:** 1 Department of Neurology, University of California-Irvine (UCI) School of Medicine; Children’s Hospital of Orange County (CHOC), CHOC Children’s Research Institute, Neuro-Oncology and Stem Cell Research Laboratory (NSCL), USA; 2 University of California-Irvine School of Social Ecology, Social and Behavioral Sciences Gateway, USA; 3 AB Sciex, Inc., Danaher Corporation, USA

**Keywords:** Traditional Chinese Medicine (TCM), Cancer niche as garbage disposal machine, Cancer metabolism

## Abstract

Cancer epidemic led to worldwide to search for a new “game changer” concept to govern cancer research and cancer treatment. Western medicine-based cancer research has been extending the impasse without resolution in sigh for improving survival of patients with solid malignant tumors in the last four decades due to heterogeneity in cancer tissues. Such a deadlock charts a course to learn lessons from the developing countries, directly or indirectly to complement the exhausted Western medicine. We propose a new concept of “Cancer niche as a garbage disposal machine” with implications of traditional Chinese medicine-mediated restoration of normal balance between body and disease to bring the fight against cancer under control.

## Background

“A total of 1,762,450 new cancer cases and 606,880 deaths from cancer are expected to occur in the US in 2019”. Published based on data in the most recent (2006 – 2015), “the rate of new cancer diagnoses decreased by about 2% per year in men and stayed about the same in women. The cancer death rate (from year 2007 to year 2016) declined by 1.4% per year in women and 1.8% per year in men” [1]. “An estimated 1,762,450 cancers will be in diagnosed in 2019, which equals to more than 4,800 new cases each day.” “22% of deaths in the US in 2016 were from cancer, making it the second leading cause of death after heart disease in both men and women.” In the same report, “Cancer is the second most common cause of death among children ages 1 to 14 years in the US, after accidents. In 2019, an estimated 11,060 children in this age group will be diagnosed with cancer and 1,190 will die from it. Leukemia accounts for almost a third (28%) of all childhood cancers, followed by brain and other nervous system tumors (26%).”

From the time when US President Richard Nixon announced, “War on cancer” up to the US President Barrack Obama launched “ ‘Moonshot’ to Cure Cancer, “ cancer researcher’s intelligence and ambitious aims too often collide with an astonishing lack of subtlety and aesthetic judgment of cancer complexity [2]. This scientific reductionism school of cancer-focused concepts has dominated in cancer research for the last century, with a representative of the Hallmarks of Cancer as treatment principles [3]. Treatment strategies based on chasing the cancer hallmarks did not change the clinical outcomes, such as cancer deaths in solid tumors. Billions of dollars have been poured on to cancer-focused research, including current cancer genome projects (Li SC, et al. [4]), however; little progress has been made towards treating malignant cancers. Cancer cells are still killing patients upon the late stage, as no medical methods can yet solve cancer problems. So, what is the solution?

One of the bottlenecks is to prevent the distant metastasis of cancer that causes an increasing number of cancer-related deaths. However, the potential mechanisms of metastasis and candidate biomarkers are still to be elucidated, although multiple models have been established, but with despaired results [5,6].

For example, as we all know, breast cancer in situ is usually not fatal to patients’ lives, however; advanced breast cancer with lymph-nodes and/or distant metastasis tends to cause life-threatening outcomes for patients [7]. It has been speculated that “since the etiology of breast cancer metastasis is largely unclear and there are no defined means to predict the metastasis and recurrence of breast cancer in clinical practice, it is urgent to explore further the potential mechanisms of metastasis and biomarkers to monitor recurrence of breast cancer.”

Some thought-leaders came up with an analogy: cancer is like a seed, the body is like soil. Whether this seed will sprout and grow up will depends on the soil, not on the seed. Similarly, no matter what kind of the seeds are planted, if the soil is not suitable, it will never sprout and grow. How to improve this soil to prevent the seed from sprouting? This is the subject of current research. Cancer cells are brilliant, fitting well to their niche the tissue microenvironment. For example, the most suitable environment for liver cancer cells to grow is the liver. If the liver is thoroughly exhausted, cancer cells will disperse to other organs. When a patient obtains a cancer-free liver via organ transplantation, the liver cancer cells return to the transplanted liver. In reality, liver cancer cells are a cousin of healthy liver cells and they co-habit on the liver niche. How then, can researchers engineer a new liver that prevents the occurrence of liver cancer cells? Consolidating the tissue barriers (the boundary of tissue microenvironment) can be accomplished via normalization of the host immune system, which conjures up hope for immunotherapy of cancer like CAR-T platform [8]. The pairwise analysis of single-cell transcriptome profiling of tumor tissue and neighboring stromal and immune cells provides insight into “intratumoral heterogeneity, which is shaped by the tumor cells and immune cells in the surrounding microenvironment” [9]. However, this approach is not applicable in the clinic, as this is only a one-time point, and does not fully capture features of the cancer dynamic.

## Limitation of Current Cancer Research

Features of the cancer dynamic have been fully captured and illustrated in mouse models of cancer. The use of animal models for human tumor testing, involving the animal’s immune system, is difficult: the animal’s immune system repels human tumor cells. Therefore, many mouse tests involve mice with impaired immune systems. Until recently, some companies began to develop a humanized mouse model in which the mouse’s immune system can tolerate human tumor cells. At present, such a mouse model is costly. The cost rises higher due to current main-stream research obsession with “statistically significant data, which requires hundreds even a thousand of humanized mice”. Such repetitiveness of scientific experiments and statistical data is not meaningful in dealing with individual patients, who demand “personalized medicine “or “precision medicine”–Notaone-size-fits-all treatment scheme.

The precision medicine approach shows why multiplicity in large scale studies using, for example, microarray genomic data, led to misclassification, based on irrespective of the p-value [10–12]. Alarmed by this big-data approach, the American Statistical Association has launched the online home for the publications of “Introduction: Special Issue on Data Science”.

DOI: 10.1080/00031305.2018.1438699), alerting that irrespective of the p-value or “P < 0.05” might not mean what you think - *“A* statistically significant test result (P ≤ 0.05) means that the test hypothesis is false or should be rejected. A P value greater than 0.05 means that no effect was observed.” “Application and interpretation of statistical evaluation of relationships is a necessary element in biomedical research. Statistical analyses rely on P-value to demonstrate relationships. The traditional level of significance, P<0.05, can be negatively impacted by small sample size, bias, and random error, and has evolved to include interpretation of statistical trends, correction factors for multiple analyses, and acceptance of statistical significance for P>0.05 for complex relationships such as effect modification.” A modification of the Bayes factor based on parametric likelihoods in the standard technique to compute p-values for hypothesis testing came out “for the nonparametric setting in asymptotic approximations to the proposed procedures, showing that the developed procedures have excellent operating characteristics for one-sample and two-sample data analysis” [13].

## Cancer is a Moving Target

Magic Bullet? Target Therapy? Gene Therapy? Reasoning Design? Cocktail Drugs for Signaling Pathways? Immunotherapy? Cancer Genome? Moon-Shot? AI (Artificial Intelligence) Medicine? Approach to Tumor Microenvironment?

Cancer research has brought up many buzzword methods in the last century, including Magic bullet. Target therapy. Gene therapy. Reasoning Design of drugs. Cocktail drugs for Signaling pathways. Immunotherapy (Li S, et al. [8]), Cancer genome (Li SC, et al. [14]), Precision oncology, single-cell approach of cancer (Li SC, et al. [5,6]), cancer subclonal evolution landscape (ILi SC, et al. [15]), CRISPR–Cas9-based genome editing of cancer treatment, stem cell therapy of cancer (Li SC, et al. [16]), Moon-shot of cancer, and Artificial Intelligence of Medicine (AI Medicine), just to name a few. The following examples illustrate that some of these strategies did not prevail over cancer heterogeneity and complexity.

Since the concept of a “magic bullet” for targeted drugs was envisioned about a century back, the approvals of brentuximab anti-CD33-ADC vedotin (in 2011) (antibody-drug conjugate (ADC)) anti-HER2 ADC trastuzumab emtansine (in 2013) and Mylotarg CD33-Calicheamicin ADC (in 2017) have spurred the development of ADCs with over 250 clinical trials and more than 60 distinct ADC molecules currently in development [17]. Results from over 250 ADC clinical trials show that “Despite this revolutionary growth, the low therapeutic index or limited safety window of ADCs has posed a significant challenge in achieving success for the treatment of solid tumors.” Overall improvement of the therapeutic index has been achieved by current strategies for increasing tumor cytotoxicity while decreasing drug toxicity.

“Prioritization of therapeutic cancer targets using CRISPR–Cas9 screens CRISPR–Cas9-based genome editing provides high specificity and produces penetrant phenotypes as null alleles can be generated” [18]. Can we cure cancer by rewriting DNA with CRISPR–Cas9-based genome editing? “In a screen of 324 human cancer cell lines and utilizing a systematic target prioritization framework, they found that the Werner syndrome ATP-dependent helicase be a synthetic lethal target in the tumor from multiple cancer types with microsatellite instability, providing a new target for cancer drug development.” – This data-driven framework derived from 30 cancer types can prioritize candidates for cancer therapeutics, as validated by “cell fitness effects with genomic biomarkers and target tractability for drug development to systematically prioritize new targets in defined tissues and genotypes”. The principles of identifying the initial stages of portfolio-based cancer drug targets remains to be elucidated in clinical trials.

“Personalized medicine and immunotherapy are changing the landscape of cancer diagnosis and treatment. Traditionally, the one-biomarker-per-drug model has been driving the development of single companion diagnostic tests to inform patient selection for drug therapy. This simple paradigm has been questioned recently, as our understanding of the complexity of tumor development has advanced—a single biomarker may provide insufficient information for accurate patient stratification. Importantly, the development of multiple tests for the same biomarker, often for the same indication, has further complicated the analysis. A classic example is the case of PD-L1, for which there are currently five different immunohistochemistry assays for use with five different PD-1/PD-L1 monoclonal antibody therapies” (Sacha Gnjatic, Ph.D.; David Rimm, MD-PhD; Houssein Abdul Sater, M.D.). In fact, it appears that cancer genome project in pure in silico practice of interest for analysis of big data can help find some leads; however, given the nature of the commercially available DNA sequencers and bioinformatics tools (based on the known facts) and the databases derived from the heterogeneity of cancer tissues and the diversified patients pools in the public domains, its value has been diminished [4]. These cancer driver biomarker leads should have been validated with the patients’ specimens, unfortunately, however, they have not yet been done. Simply speaking, cancer is a moving target in term of molecular markers and gene mutations. Targeting molecular mutations unlikely can cure cancer.

## Alternative aspect of cancer research

An alternative to cancer gene mutations, the tumor microenvironment concept has gained traction through time, in light of cancer stem cell hypothesis. “Pediatric origin of cancer stem cell hypothesis holds great promise and potential in adult cancer treatment; however; the road to innovation is full of obstacles as there are plenty of questions left unanswered. First, the key question is to characterize the nature of such stem cells (concept). Second, quantitative imaging of pediatric stem cells should be implemented (technology). Conceptually, pediatric stem cell origins of adult cancer are based on the notion that plasticity in early life developmental programming progresses local environments to cancer. Technologically, such imaging in children is lacking as all imaging methods are designed for adult patients. We postulate that the need for quantitative imaging to measure space-time changes of plasticity in early life developmental programming in children may facilitate and necessitate research and development of the imaging technology. Such quantitative imaging of pediatric origin of adulthood cancer will help develop a spatiotemporal monitoring system to determine cancer initiation and progression. Clinical validation of such speculative hypothesis-that cancer originates in a pediatric environment-will help implement a wait-and-watch strategy for cancer treatment” [5,6].

“Targeting the tumor microenvironment (TME) through which cancer stem cells (CSCs) crosstalk for cancer initiation and progression, may open new treatments different from those centered on the original hallmarks of cancer genetics thereby implying a new approach for suppression of TME driven activation of CSCs. Cancer is dynamic, heterogeneous, evolving with the TME and can be influenced by tissue-specific elasticity. One of the mediators and modulators of the crosstalk between CSCs and mechanical forces is miRNA, which can be developmentally regulated, in a tissue- and cell-specific manner. Here, based on our previous data, we provide a framework through which such gene expression changes in response to external mechanical forces can be understood during cancer progression. “Recognizing the ways mechanical forces regulate intracellular signals came up with applications in cancer stem cell biology. For example, TME-targeted pathways shed new light on strategies for attacking cancer stem cells with fewer side effects than traditional gene-based treatments for cancer, requiring a “watch-and-wait” approach. This approach attempts to address both normal brain microenvironment and tumor microenvironment as both works together, intertwining in pathology and physiology - a balance that needs to be maintained for the “watch-and-wait” approach to cancer. Connecting the subjects of tissue elasticity, tumor microenvironment, epigenetics of miRNAs, and stem-cell biology is very relevant in cancer research and therapy, unifying” apparently separate entities in a complex biological web in a realistic and practical manner can bridge basic research with clinical application” [19]. Such integration of interdisciplinary research gave birth of a new technology, namely AI Medicine.

Collectively, AI Medicine has emerged as a transformative tool to characterize heterogeneous cancer; nevertheless, translating the wealth of data available into biological insight remains challenging for clinical benefits. AI Medicine presents exciting opportunities for data integration of the diversity of technologies, modalities, and systems underlying new datasets. Furthermore, new experimental and computational methods may allow for the integrated learning of cellular states, as well as an approach to ‘align’ shared subpopulations across health-related datasets. Holistic care of cancer, an emerging comprehensive care platform of cancer, is close to such one-of-a-kind approaching to the tumor microenvironment. This platform integrates those care methods, including molecular medicine against cancer cells, nutrition management, and lifestyle.

## The Hypothetical Concept: Cancer Niche as Garbage Disposal Machine: Implications for New Treatment of Cancer

Structure-function coordination is a principle of biology. Irregular coordination of a structure-function axis made of cancer leads to malignancies. Strategies for restoration of the normal axis are designed to target cancer that causes deaths. Cancer is a structure in the human body. If the body is compared to a house, each organ correspondingly a separate room with a garbage container for sanitation in each – similarly, each organ has a garbage niche for disposal of diseased cells. The immune barrier (boundary) solidifies homeostasis. Artificial engineering of T-cells as in CAR-T, which are hard to manage in equilibrium – break down the barrier that led to detrimental effects [8]. CAR-T cells are subjected to the principle of biological control in the ecosystem as for the introduction of Australian rabbits as illustrated below.

“Arid Australia has the world’s worst modern mammalian extinction record, largely attributable to competition from introduced herbivores, particularly European rabbits (Oryctolagus cuniculus) and predation by feral cats (Felis catus) and foxes (Vulpes vulpes). The biological control agent rabbit hemorrhagic disease virus (RHDV) was introduced to Australia in 1995 and resulted in dramatic, widespread rabbit suppression.” “Funding for species conservation is insufficient to meet the current challenges facing global biodiversity, yet many programs use expensive single-species recovery actions and neglect broader management that addresses threatening processes.” “Conservation programs that take big-picture approaches to address threatening processes over large spatial scales should be prioritized to maximize the return from limited conservation funding. Further, these should be coupled with long-term ecological monitoring, a critical tool in detecting and understanding complex ecosystem change” [20].

“Restoring Balance: Using Exotic Species to Control Invasive Exotic Species” [21]. “Invasive species threaten natural habitats worldwide, and active human management is required to prevent invasion, contain the spread, or remediate ecosystems following habitat degradation. One powerful technology for invasive species management in sensitive habitats is biological control, the use of carefully selected upper-trophic-level organisms that utilize the exotic pest as a resource, thereby reducing it to less harmful densities. Many in the conservation biology community view this pest-management technology as a high-risk enterprise because of potential collateral damage to nontarget species. The potential benefits arising from successful biological programs are reduced pesticide use, significant pest suppression, and a return to environmental conditions like those observed before the arrival of the pest. Biological control as a pest-management strategy has limitations: some pest species may not be suitable targets for biological control because natural enemies may not be sufficiently host-specific and may pose a threat to nontarget organisms. In some instances, substantial effects on nontarget species have occurred because generalist natural enemies established as part of a biological control program heavily utilized other resources in addition to the target pest. To minimize nontarget impacts, they managed the regulations governing releases of natural enemies are becoming more stringent, as evidenced in New Zealand and Australia. Voluntary codes of good practice are being advocated by the Food and Agriculture Organization to promote wide adoption of safety measures, which, if followed, should result in the selection of agents with high levels of host and habitat fidelity. In another example. Biological control programs in support of conservation have traditionally targeted weed species that threaten natural areas. More recently, exotic arthropod pests that compete with native wildlife or damage native plants have become targets of conservation-oriented biological control programs. Extension of biological control to new targets of conservation importance, such as invasive aquatic invertebrates and pestiferous vertebrates, is warranted. In many instances, once conventional prevention, containment, and eradication options have been exhausted or deemed infeasible, carefully orchestrated biological control programs against appropriately selected targets may be the only feasible way to control invasive species affecting communities under assault from exotic species.”

Borrowing from the metaphor above, researchers came up with a concept of spatiotemporal switching control of cancer with TME as an ecosystem [5,6]. Some examples can be used to illustrate such control switching mechanism. “Identification of a mesenchymal progenitor cell hierarchy in adipose tissue) indicated that fatty tissue could expand in two ways: through increases in the size of individual adipocytes or through increases in the number of adipocytes” [22]. The former process promotes metabolic disease, and the latter protects against it. Merrick et al. used single-cell RNA sequencing to define the hierarchy of mesenchymal progenitor cells that give rise to adipose tissue in mice and humans (see the Perspective by Chau and Caw thorn). They found that “progenitor cells expressing a protein called DPP4 to give rise to two distinct types of preadipocytes in response to different signals. The DPP4 progenitors reside in a fluid-filled network of collagen and elastin fibers surrounding adipose tissue. In principle, therapeutic interventions that increase progenitor cell differentiation into adipocytes could ameliorate metabolic disease.” The same idea can be used for cancer treatment through managing TME.

Another example of the ecosystem-like management of disease has been amyotrophic lateral sclerosis (ALS), which has some aspects in common with multiple myeloma, as both are protein misfolding diseases, with ALS-specific mutations within the profilin 1 (PFN1) gene [23]. “ALS-associated ubiquitinated PFN1 is insoluble aggregates that in many cases contain the ALS-associated protein TDP-43, which is ubiquitinated, hyperphosphorylated, and N-terminally truncated TDP-43 involved in toxicity” [24]. “Multiple myelomas are a cancer of plasma cells, a type of white blood cells responsible for producing antibodies. The abnormal plasma cells then produce abnormal antibodies, eventually crowding out healthy plasma cells and interfering with the work that normal antibodies do. As the healthy plasma cells in the bone marrow are depleted by the cancer cells, multiple myeloma causes symptoms, including increased infections, anemia, and bone pain. Myeloma often goes undetected until the symptoms are severe, and eventually lead to several painful compression fractures before diagnosis, by which point, the cancer cells composed 70 percent of the bone marrow with Patient Jill.” “Current treatment for multiple myeloma includes bone marrow depletion followed by stem cell recovery. It starts with highly toxic chemotherapy that destroys all immune cells, followed by a stem cell transplant to the bone marrow where healthy stem cells begin the task of rebuilding the immune system. Thus, with virtually no immune system, the patient is susceptible to every kind of infection: even the slightest cold can be a danger. Until the stem cells finish their work, the patient is confined to isolation, sometimes for weeks or months.” With the improved technology, we published a technology platform to track the multiple myeloma for stage-specific treatment, which minimized the adverse effects of chemotherapy and improved clinical outcomes [25]. Nonetheless, little is known about the balance of cancer ecosystem, including TME, immune restoration, and cancer subclonal evolution landscape. A new concept is desperately needed.

A hypothetical concept can be postulated for a cancer niche as a metaphorical garbage disposal machine. Such a garbage disposal machine implicates new treatments of cancer. Specifically, this garbage disposal machine is present in each organ of the body – a mechanism to keep cancer cells alive but within the garbage container. The cancer niche as a disposal garbage container is essential for sanitation of the body. Removal of such a disposal garbage container, such as the removal of the primary tumor, may break the barrier of the tumor, initiating the cancer cells to metastasis. Keeping such a cancer niche, but monitoring and maintaining it in dormancy by blocking it from contacting with wake-up calls or signals (cytokines or hormones) Li SC, et al. [14,15], may be a solution to prevent cancer from damaging the body. That is because both healthy cells and cancer cells share the same signaling pathways and resources [26]. Metabolites accumulation in patients can trigger the production of inflammatory molecules, which in turn trigger cell outgrowth or overreaction (pathogenesis). Cancer metabolism generates high heat, which is the base for the CT scan to distinguish them from the healthy cells (low heat). One component of the garbage disposal is proteasome regulators, and machinery systems, including that ZFAND, enhances multiple 26S activities and overall cellular protein breakdown [27]. The mechanism of action is coordinated within the induction of autophagy-lysosome and ubiquitin-proteasome systems [28]. E.g., bortezomib, which inhibits the major peptidase sites in the proteasome’s 20S core particle proteasome, improved the outcomes of the treatment of multiple myeloma [29].

“Cancer cells can shift their energy production to resist proteasome inhibitors. Fortunately, Peter Tsvetkov’s laboratory at Whitehead Institute identified a drug that makes resistant cells susceptible to proteasome inhibitors, combatting the cells’ attempt to sidestep the treatment.” All these indicate that modulating the organ’s garbage disposal can regulate cancer cell growth.

## The TCM-modulated Machinery of Garbage Disposal

In Nature, a healthy forest sustains ecosystems over time and space, which can provides value to living beings by frequently trimming or down-regulating the growth of certain trees or bushes or grasses in order to keep it healthy. The loss of a tree species however can have cascading harmful effects on the forest ecosystem and on the benefits provided to human populations. The body manages the garbage disposal system designed to trim down the outgrowing cancer cells that cause cancer related death. Body management of health is done by the immune system. “For decades, even centuries, the developing world has looked to the West, directly or indirectly, to chart a course and learn lessons. The time may be coming for U.S. health care to look eastward to find some valuable lessons.” (David J. Cook, MD, MHA & Paula Li, Ph.D.) Such a paradigm shift may reveal how cancer threats have been unaddressed for decades and may be addressed by TCM (Traditional Chinese Medicine), with the Eastern philosophy of healthcare management.

What the TCM treatments do is harness the body’s own immune system to fight diseases – the principle of TCM is 扶正祛邪 (strengthen the rightness of the body thereby getting rid of evil). The consolidated immune system thus takes the responsibility to detect and treat tumors. The TCM approach takes up the overall balance of rigorous cross-disciplinary integration of physiology and pathology. For example, “Weight loss and tissue wasting often occur in pancreatic cancer. Analyses of human and mouse data revealed a mechanism behind these events and raise the question of whether tissue wasting affects cancer survival rates” [30]. J. Matthias Löhr pointed out: “Cachexia, the term used to describe the cancer-linked symptom of severe weight loss, has been recognized since the time of the ancient Greek physician Hippocrates. It is often a hallmark of cancers originating in the gut system and might manifest in changes such as loss of fat (adipose) tissue or skeletal-muscle wasting, which could arise if the body is using up the nutrients stored in such tissues. Cachexia is particularly common in people who have a type of cancer called pancreatic ductal adenocarcinoma. The mechanisms driving cachexia are not the same in all tumor, but whether there are different types of cachexia depending on the tumor type or the stage of cancer at which weight loss occurs remains to be determined.” Specifically, “two key mechanisms thought to drive cachexia are the breakdown of molecules in a process called catabolism, and inflammation, which is controlled by the body’s immune system. The pancreas secretes digestive enzymes that break down complex, calorie-rich food to provide the components needed for tissue growth and maintenance; this catabolism-supporting function is known as its exocrine role. Exocrine-system impairment causes malnutrition that can lead to life-threatening tissue wasting. However, the degree to which pancreatic exocrine-system abnormalities contribute to human cachexia was unknown” [31]. The pancreas secretes enzymes into the small intestine that aid the breakdown of food, enabling nutrients to be absorbed. Abnormalities in this process can lead to weight loss and adipose tissue wasting, which occur early in pancreatic cancer. Mice with pancreatic tumor had lower blood glucose levels, increased levels of adipose-tissue wasting, and a decreased survival rate compared with control mice that did not have a pancreatic tumor. “If you are improving this wasting effect that we see in pancreatic cancer, you would expect that you would also improve survival” [30]. Aligned well with this observation, TCM can help metabolism to exert cardioprotective activities Zhou JM, et al. [32]. TCM modulations can elaborate the mechanisms of “sexual hormone levels, the expression of ER and PR, hemorheology, free radical activity and lipid peroxidation, VEGF and BFGF, cell proliferation activities, anti-apoptosis gene BCL-2, promoting apoptosis gene Bax, ERK, and tumor suppressor gene”, thereby controlling mammary gland hyperplasia that is related to breast cancer [33].

In summary, the most important aspect of TCM-mediated-modulations lies in the prevention of diseases by using the naturally existed conditions and components. For example, “Rather than seeking to design and synthesize new proteins rationally, piece by carefully calculated piece — as many protein chemists have tried and mostly failed to do — Nobel Laureate Frances Arnold approach lets basic evolutionary algorithms do the work of protein composition and protein upgrades by taking naturally existed ingredients - from the protein frameworks exist in Nature Natalie Angier.

May 28, 2019, New York Times). “Dr. Arnold won the Nobel Prize for developing such a technique called directed evolution, a way of generating a host of novel enzymes and other biomolecules that can be put to any number of uses — detoxifying a chemical spill”. In reality, TCM most likely exerts its impact by directly-induced evolution of human body physiology to adapt the microenvironment, as the body senses and responds to the changes. For example, a Taiwan-population-based cohort study of 386 patients in treatment arm with 386 in control arm shows that traditional Chinese medicine [TCM] guided Chinese herbal medicine [CHM] therapy was associated with reduced mortality among patients with pancreatic cancer. Specifically, “CHM users had a lower hazard ratio of mortality risk (adjusted HR = 0.67, 95% CI = 0.56-0.79). Those who received CHM therapy for more than 90 days had significantly lower hazard ratios of mortality risk than non-CHM users (90- to 180-day group: adjusted HR = 0.56, 95% CI = 0.42-0.75; > 180-day group: HR = 0.33, 95% CI = 0.24-0.45), The survival probability was higher for patients in the CHM group.” [34]. The mechanisms remain to be elucidated.

## Perspective

The concept cancer niche as a garbage disposal machine implies TCM-mediated balance of body-disease for treatment of cancer. We need to make peace with cancer by sharing the resources of the body and environment. AI-based research will develop a mathematical framework to explain how the cancer garbage disposal represents an organ of dynamic cell debris, in an aging body, with predicted changes through a lifetime. Experiments will verify the framework model to guide healthcare.
